# Effects of different temperatures of carbohydrate-protein-containing drinks on gastric emptying rate after exercise in healthy young men: randomized crossover trial

**DOI:** 10.1186/s40101-022-00311-2

**Published:** 2022-10-25

**Authors:** Kyoko Fujihira, Masaki Takahashi, Kei Shimamura, Naoyuki Hayashi

**Affiliations:** 1 Japan Society for the Promotion of Science, Tokyo, Japan; 2 Institute for Liberal Arts, Tokyo Institute of Technology, Tokyo, Japan; 3 Department of Social and Human Sciences, Tokyo Institute of Technology, Tokyo, Japan; 4 Faculty of Sport Sciences, Waseda University, Saitama, Japan

**Keywords:** Drink temperature, Exercise, Gastric emptying, ^13^C breath test

## Abstract

**Background:**

The present study examined the effects of different temperatures of carbohydrate-protein-containing drinks after exercise on the subsequent gastric emptying rate in healthy young men.

**Methods:**

Twelve healthy young men completed two, 1-day trials in random order. In both trials, the participants completed intermittent cycling exercise for 20 min, consisting of a 120% heart rate peak for 20 s, followed by 25 W for 40 s. Participants consumed 400 mL of carbohydrate-protein-containing drink (0.85 MJ) at 4 °C (EX + 4 °C) or 60 °C (EX + 60 °C) over a 5-min period after exercise. The participants sat on a chair for 2.5 h to measure their gastric emptying rate using the ^13^C-sodium acetate breath test. Subjective feelings of gastrointestinal discomfort and appetite were measured using a visual analog scale. Interstitial fluid glucose levels after drinking were measured using a continuous glucose-monitoring device.

**Results:**

The percentage excretion of ^13^CO_2_ tended to be higher at EX + 60 °C than at EX + 4 °C from the start of the test until 30 min after drink ingestion (5.7 ± 0.5 vs. 6.5 ± 0.4%dose/h for the EX + 4 °C and EX + 60 °C trials, respectively; effect sizes [ES] = 0.277, *p* = 0.065). The time of maximum ^13^CO_2_ emissions per hour (Tmax-calc) and the time of half ^13^CO_2_ emissions per hour (T_1/2_) did not differ between trials. Subjective gastrointestinal discomfort was lower at EX + 60 °C compared to EX + 4 °C (ES = 0.328, *p* = 0.041). There were no significant differences in interstitial fluid glucose levels between the different temperatures of carbohydrate-protein-containing drinks after exercise (*p* = 0.698).

**Conclusions:**

Consumption of warm carbohydrate-protein-containing drinks after exercise may accelerate gastric emptying in the very early phase and may reduce gastric discomfort.

**Trial registration:**

University Hospital Medical Information Network, UMIN000045626. Registered on June 10, 2021.

## Introduction

The position paper on nutritional supplementation for exercise shows that supplementation of carbohydrate and protein after exercise improves muscle protein synthesis [1]. Carbohydrates and proteins ingested after exercise are released from the stomach, following which carbohydrates and proteins are broken down by enzymes into monosaccharides and amino acids/dipeptides/tripeptides and absorbed in the small intestine to help muscle recovery. However, previous studies have shown that gastric emptying is delayed immediately after exercise [2, 3].

To date, three laboratory-based studies have examined gastric emptying after exercise drink ingestion. Studies have reported lower gastric emptying rates when carbohydrate-protein or carbohydrate drinks were consumed 5 min after intermittent cycling or resistance exercise than when consumed at rest or 30 min post-exercise [2, 3]. However, gastric emptying rate did not change after mild- to moderate-intensity cycling exercises [4]. A meta-analysis of acute exercise and gastric emptying suggests that exercise intensity, mode, and duration, as well as the volume and composition of the drink consumed, affect the variability of gastric emptying during and after acute exercise [5]. Although studies on exercise have not been examined, studies at rest have identified that drink temperature is one of the factors influencing gastric emptying [6–9].

Drink temperature changes gastrointestinal motility, such as gastric contractions, via changes in intragastric temperature [10–13]. Previous studies examining the effects of warm drink ingestion on gastric motility showed that consumption of warm drink (50–60 °C) increased gastric contractions compared with consumption of cold or body-temperature drinks (2–37 °C) [10–13]. Peristaltic motions of the stomach propagate due to induction by slow waves from the cardia to pyloric antral area and controlled gastric motility and gastric emptying. A study that examined changes in peristalsis with increasing temperature in smooth muscle from guinea pigs showed that elevating the temperature from 24 to 42 °C increased the frequency and maximum rate of rising of the upstroke phase of slow waves [14]. Indeed, a study has shown that gastric emptying is faster in warm drink trials than in colder drink trials [7]. The effect of drink temperature on gastric emptying may differ from that at rest because the core temperature is temporarily elevated during exercise [15]. However, to our knowledge, no studies have examined the effects of different temperatures of carbohydrate-protein-containing drinks on gastric emptying after exercise. As gastric emptying is associated with subsequent nutrient absorption and appetite, investigating the effects of post-exercise drinking temperature on gastric emptying may provide new insights into post-exercise nutritional strategies.

Thus, the purpose of this study was to investigate the effects of different temperatures of carbohydrate-protein-containing drink after exercise on the gastric emptying rate in healthy young men. We hypothesized that consuming a carbohydrate-protein-containing drink at 60 °C would result in faster gastric emptying compared to the consumption of a carbohydrate-protein-containing drink at 4 °C.

## Methods

### Participants

The Ethics Committee of Tokyo Institute of Technology, Japan, approved this study (approval number: 2021059). This study was registered in the UMIN Clinical Trial Registry (UMIN: 000,045,626) and conducted in accordance with the Code of Ethics of the World Medical Association (Declaration of Helsinki). A total of 12 young, healthy males with an exercise routine of at least 30 min per session, at least once a week, provided written informed consent to participate in this study. The physical characteristics of the participants were as follows: age: 23.0 ± 1.3 years; height: 1.73 ± 0.06 m; body mass: 66.5 ± 13.6 kg; and body mass index: 22.1 ± 4.3 kg/m^2^. The physical and descriptive characteristics of the participants are shown in Table 1. The exclusion criteria were as follows: smokers, major illness, digestive symptom, medications that affect gastric motility and appetite, food allergies to the food in the test meal, restrained eaters, or body masses unstable for at least 3 months before the study.

**Table 1:** The characteristics of the participants

Characteristic	Means ± SD
Age (years)	23.0 ± 1.3
Height (m)	1.73 ± 0.06
Body mass (kg)	66.5 ± 13.6
Body mass index (kg/m^2^)	22.1 ± 4.3

*N* = 12; data are means ± SD

### Preliminary test session

Participants visited the laboratory prior to the main trials for preliminary assessment. Height and body mass were measured using a body fat analyzer using the impedance method (Inbody230, InBody Japan Inc., Germany), and BMI was subsequently calculated. To determine individual target work rates, a ramp incremental exercise test using a cycling ergometer (COMFORT5, Johnson Health Tech Japan, Japan) was performed at least 6 days prior to the experiment. This test included 2 min of baseline rest in the sitting position, followed by 2 min of baseline exercise at 25 W and incremental ramp exercise at 15–20 W/min, still within the individual’s tolerance limit. The participants were instructed to maintain a pedaling frequency of 60 rpm. Ratings of perceived exertion (RPE) were recorded at the end of each stage during both exercise tests using Borg’s scale [16]. The criteria used to confirm a maximum value included two or more of the following: (1) participants could not maintain 50 rpm despite maximal exertion and (2) RPE ≥ 19. Based on the maximum heart rate and work rate measured in the preliminary test session, individual target work rates at 120% heart rate (HR) peak were determined (348 ± 51 W).

### Main session protocol

The participants underwent two, 1-day laboratory-based trials in random order: (1) 400 mL of carbohydrate-protein-containing drink at 4 °C after exercise (EX + 4 °C) and (2) 400 mL of carbohydrate-protein-containing drink at 60 °C after exercise (EX + 60 °C). The interval between trials was at least 6 days. All participants were asked to maintain their normal eating habits between trials and to refrain from vigorous exercise and alcohol intake for 24 h before each trial. Participants attached the FreeStyle Libre (71,533–01, Abbott, USA) to the back of their upper arm on the day of the experiment and up to 3 days before the experiment to stabilize the continuous glucose monitoring device. At 24 h before the first trial, the participants weighed and recorded all dietary intakes, and subsequently replicated these dietary intakes in the 24 h preceding the second trial. Food diaries were analyzed using a software program (Excel Eiyou-kun, ver 9.0, Kenpakusha, Japan) to determine energy intake and macronutrient content. On each trial day, the participants visited to the laboratory at 08:45 after a 10-h overnight fast. Participants were allowed to drink only one, 200 mL glass of water no later than 2 h prior to each trial. After measuring their weight and body composition on the day of the study, the participants were seated in a chair in a laboratory room for 5 min where the temperature and humidity were maintained at 22.1 ± 1.5 °C and 26.8 ± 4.6%, respectively. After 2 min of warm-up cycling at 25 W, the participants followed by 20 sets of moderate-intensity interval exercises, in which one set consisted of 120% HRpeak for 20 s and 25 W for 40 s as a recovery. The duration of the exercise was determined by preliminary tests and set as long as the subject could achieve. The preliminary and main exercise protocol was based on a previous study [2]. HR was measured at the end of the recovery performed at 25 W for 40 s. RPE was recorded at the end of each stage during both exercise tests using the Borg scale [16]. The participants consumed 400 mL of a nutrient drink containing of 21.2 g carbohydrate and 30 g milk protein (ZAVAS Carbohydrate-protein Drink, Meiji, Tokyo, Japan) at 5 min after exercise of either at 4 °C (58.8% protein, 0.0% fat, and 41.2% carbohydrates; 0.85 MJ; 4 °C) or 60 °C (58.8% protein, 0.0% fat, and 41.2% carbohydrates; 0.85 MJ; 60 °C). The carbohydrates in the drink were lactose and trace amounts of dextrin. The temperature of the drinks has been specified by the previous study [7]. Milk protein contains casein and whey protein, and casein is known to be less denatured by heat treatment [17]. In the previous study examining the thermal denaturation of whey protein at different temperatures, whey protein was rarely denatured at 65 °C [18], suggesting that casein and whey protein were not denatured at the temperature of 60 °C used in the present study. Temperature of carbohydrate-protein-containing drink was measured using an electric thermometer (TT-508 N, TANITA Corp., Japan). After consuming the carbohydrate-protein-containing drink, the participants were placed in a sitting position for 2.5 h. To measure digestion and absorption rates, exhaled breath samples were collected using small-volume bags (PAYLORI-BAG20: Fukuda Denshi) at 5- and 10-min intervals for 5–60 s each. To estimate glucose levels in the interstitial fluid, a continuous glucose monitoring device was used before, after exercise, every 5 min up to 60 min after exercise, and every 10 min up to 120 min after exercise. Participants completed visual analog scale (VAS) [19] before exercise and every 30 min after exercise to assess their perceptions of appetite and feelings of physical condition. The heart rate was monitored continuously from before exercise to 2.5 h after exercise.

### Assessment of gastric emptying rates

The rate of gastric emptying of carbohydrate-protein-containing drinks was assessed using the ^13^C-sodium acetate breath test [20]. Participants consumed a carbohydrate-protein-containing drink containing 100 mg of dissolved ^13^C-sodium acetate at 4 °C or 60 °C at 5 min after exercise. 100 mg of dissolved ^13^C-sodium acetate was weighed using an electronic scale (AXZ1204, AS ONE, Japan). ^13^C-sodium acetate breath testing correlates strongly with scintigraphy, the gold standard for measuring gastric emptying rate [20–22]. Baseline exhaled breath samples were collected using a large-volume bag (PAYLORI-BAG5 L; Fukuda Denshi, Japan), and exhaled breath samples were collected in small-volume bags (PAYLORI-BAG20; Fukuda Denshi, Japan) at 5- and 10-min intervals after the consumption of carbohydrate-protein-containing drinks. The ^13^CO_2_ ratio was measured using an isotope ratio mass spectrometer (POCone plus, Otsuka Electronics Co., Ltd., Japan). the body surface area was calculated from the body weight and height and was assumed to be 300 mmol/h per square meter of body surface. The time course of the ^13^CO_2_ recovery rate per hour and the cumulative ^13^CO_2_ recovery rate were determined. The time of maximum [^13^CO_2_] emissions per hour (T_max-calc_) and the time of half [^13^CO_2_] emissions per hour (T_1/2_) were calculated based on previous studies [3, 4]. The value of ^13^CO_2_ excretion rate in 1 h (%dose/h) was calculated to measure the difference in gastric emptying by drink temperature at each time point. The duration of the ^13^C-sodium acetate breath test was determined based on previous studies in order to calculate T_max-calc_ and T_1/2_ [7]. The value of ^13^CO_2_ excretion rate in 1 h (%dose/h) was calculated during the first 30 min, when previous studies have shown differences in gastric emptying due to drink temperature [7].

### Assessment of interstitial fluid glucose

To measure glucose levels before exercise and glucose uptake after drinking, participants wore a Freestyle Libre. The Freestyle Libre is a continuous glucose monitoring device that measures the concentration of glucose in the interstitial fluid using a less invasive method than blood sampling. Because glucose initially enters the blood and then the interstitial fluid, there may be time lags between blood glucose and interstitial fluid glucose [23]. However, there are strong correlations between blood and interstitial fluid glucose levels [24]. In the present study, the Freestyle Libre was scanned before, after, and every 5 min up to 60 min after exercise and every 15 min up to 150 min after exercise.

### Assessment of appetite score and feelings of physical condition

Participants completed the VAS [19] before and every 30 min after exercise to assess their perceptions of appetite (i.e., hunger, fullness and desire to eat, sweet, sour, fatty and salty foods) and feelings of physical condition. (i.e., “Does your stomach feel uncomfortable?” and “Do you feel exhausted?”). The verbal anchors “not at all” and “extremely” were placed at 0 and 100 mm on the VAS, respectively.

### Statistical analysis

The sample size was estimated using G*Power 3.1 [25] with reference to the data of a previous study [7] that investigated the effects of drink temperatures on gastric emptying. A sample size of 10 subjects was required to detect changes in gastric emptying with 80% power and 5% alpha level. In the present study, a dropout rate of 20% was assumed, and therefore, a sample size of 12 persons was selected. Data were analyzed using the Predictive Analytics Software for Windows (IBM SPSS Statistics 23.0, SPSS Japan, Inc., Japan). The paired t-test was used to assess the differences between trials in T_max-calc_ and T_1/2_. Repeated measures, two-factor analysis of variance (ANOVA) was used to examine differences over time between the trials the gastric emptying rate, interstitial fluid glucose, subjective appetite perceptions and subjective feelings of physical condition. Where significant trial-time interactions and trial effects were found, we used a simple main effect test to analyze these values. Partial η^2^ and 95% CI were used as effect sizes (ES). The 95% confidence intervals (95% CI) for the mean absolute pairwise differences between the trials were calculated using the t-distribution and degrees of freedom (n-1). Data were expressed as means ± standard deviation (SD). Statistical significance was set at *P* < 0.05.

## Results

### Dietary data

The mean energy intake 24 h before each trial was 8.7 ± 1.7 MJ. The participants weighed and recorded all dietary intakes in the 24 h before the first trial, and these dietary intakes were subsequently replicated in the 24 h preceding the second trial. The analyses of food diaries revealed that energy intake was constant in the 24 h preceding each trial. The energy intake equated to 32.2 ± 9.0% (74.9 ± 25.4 g/day) from fat, 50.0 ± 9.3% (260.0 ± 64.3 g/day) from carbohydrates and 15.4 ± 2.9% (80.7 ± 24.3 g/day) from protein.

### Baseline data

There were no significant differences in body mass between EX + 4 °C and EX + 60 °C trials at 0900 (i.e., pre-trial). At baseline, the VAS scores for appetite and feelings of physical condition did not differ between trials (*p* > 0.05). Interstitial fluid glucose was not different at 0900 (i.e., baseline) between the trials. The body mass, appetite score and feelings of physical condition and interstitial fluid glucose at pre-trial are shown in Table 2.

**Table 2:** Body mass, perception of appetite, feelings of physical condition, and interstitial fluid glucose value at baseline

	EX + 4 °C	EX + 60 °C	*P*
**Body mass**			
Body mass (kg)	67.2 ± 12.4	67.3 ± 12.7	0.601
**Perception of the appetite**			
Hunger (mm)	70.8 ± 10.9	69.9 ± 11.3	0.833
Fullness (mm)	22.5 ± 12.8	24.8 ± 20.0	0.726
Desire to eat (mm)	71.8 ± 8.2	67.3 ± 20.6	0.433
Desire to eat sweet foods (mm)	62.0 ± 17.2	58.9 ± 16.1	0.623
Desire to eat salty foods (mm)	59.2 ± 13.4	55.8 ± 17.7	0.493
Desire to eat sour foods (mm)	38.9 ± 16.2	45.3 ± 16.2	0.325
Desire to eat fatty foods (mm)	44.2 ± 24.0	39.7 ± 21.9	0.525
**Feelings of physical condition**			
Gastrointestinal discomfort (mm)	23.5 ± 21.8	22.8 ± 26.7	0.904
Physical exhaustion (mm)	36.7 ± 22.2	23.5 ± 21.8	0.541
**Interstitial fluid glucose**			
Interstitial fluid glucose (mg/dL)	88.6 ± 11.9	90.2 ± 11.6	0.166

*N* = 12; data are means ± SD. Data were analyzed using *t*-test. EX: 20 sets of moderate-intensity interval exercises, in which 1 set consisted of 120% maximum heart rate for 20 s and 25 W for 40 s; 4 °C: carbohydrate-protein-containing drink intake at 4 °C; 60 °C: carbohydrate-protein-containing drink intake at 60 °C

### Exercise response

**Fig. 1:**
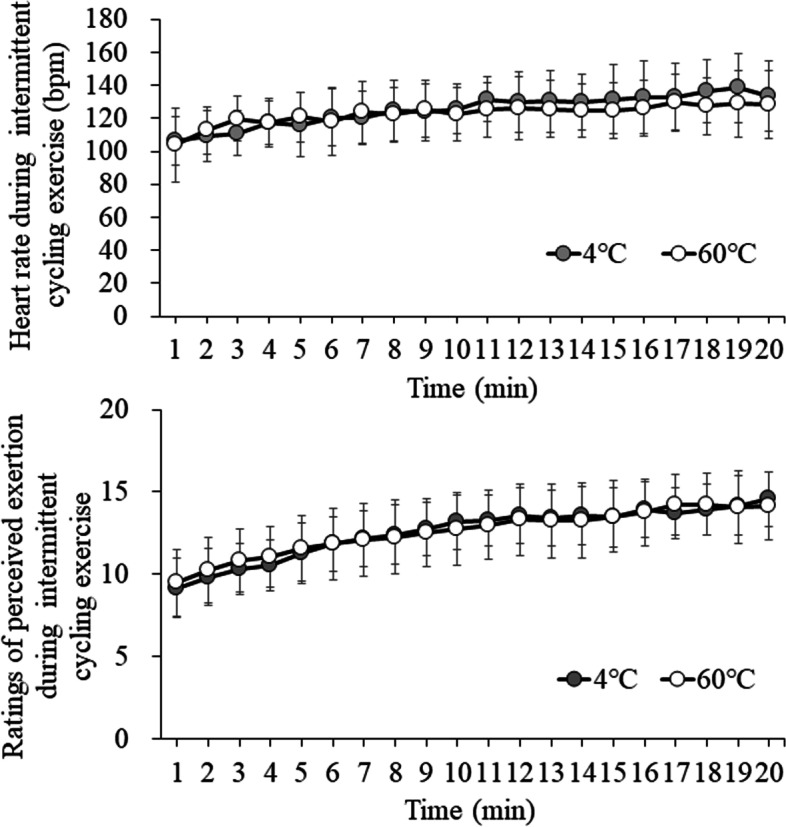
The HR and RPE changes during intermittent cycling exercise at EX + 4 °C and EX + 60 °C trials. The upper panel indicates HR, and the lower panel indicates RPE. HR and RPE were measured at the end of the recovery performed at 25 W for 40 s. Data were analyzed using two-factor ANOVA. EX: 20 sets of moderate-intensity interval exercises, in which 1 set consisted of 120% maximum heart rate for 20 s and 25 W for 40 s; 4 °C: carbohydrate-protein-containing drink intake at 4 °C; 60 °C: carbohydrate-protein-containing drink intake at 60 °C. There was a significant main effect of time (*p* < 0.001). There were no main effect of trial (*p* = 0.812) and interaction (*p* = 0.356)

The HR and RPE changes during the intermittent cycling exercise are shown in Fig. 1. The HR and RPE were measured at the end of the recovery performed at 25 W for 40 s. There were no significant differences in mean HR (125 ± 16 bpm vs. 123 ± 15 bpm for the EX + 4 °C and EX + 60 °C trials, respectively, 95% CI: − 6.8 to 11.3, *p* = 0.593) during the cycling between the trials. There were no significant differences in mean RPE (13 ± 1 vs. 13 ± 2 for the EX + 4 °C and EX + 60 °C trials, respectively, 95% CI: − 0.6 to 1.2, *p* = 0.555) during the cycling between the trials. Intermittent exercise was confirmed as moderate-intensity by RPE [26]. Also, there were no significant differences in mean HR (75 ± 6 bpm vs. 76 ± 8 bpm for the EX + 4 °C and EX + 60 °C trials, respectively, 95% CI: − 4.7 to 3.4, *p* = 0.713) from the end of the exercise to 150 min later.

### Gastric emptying rate

**Fig. 2:**
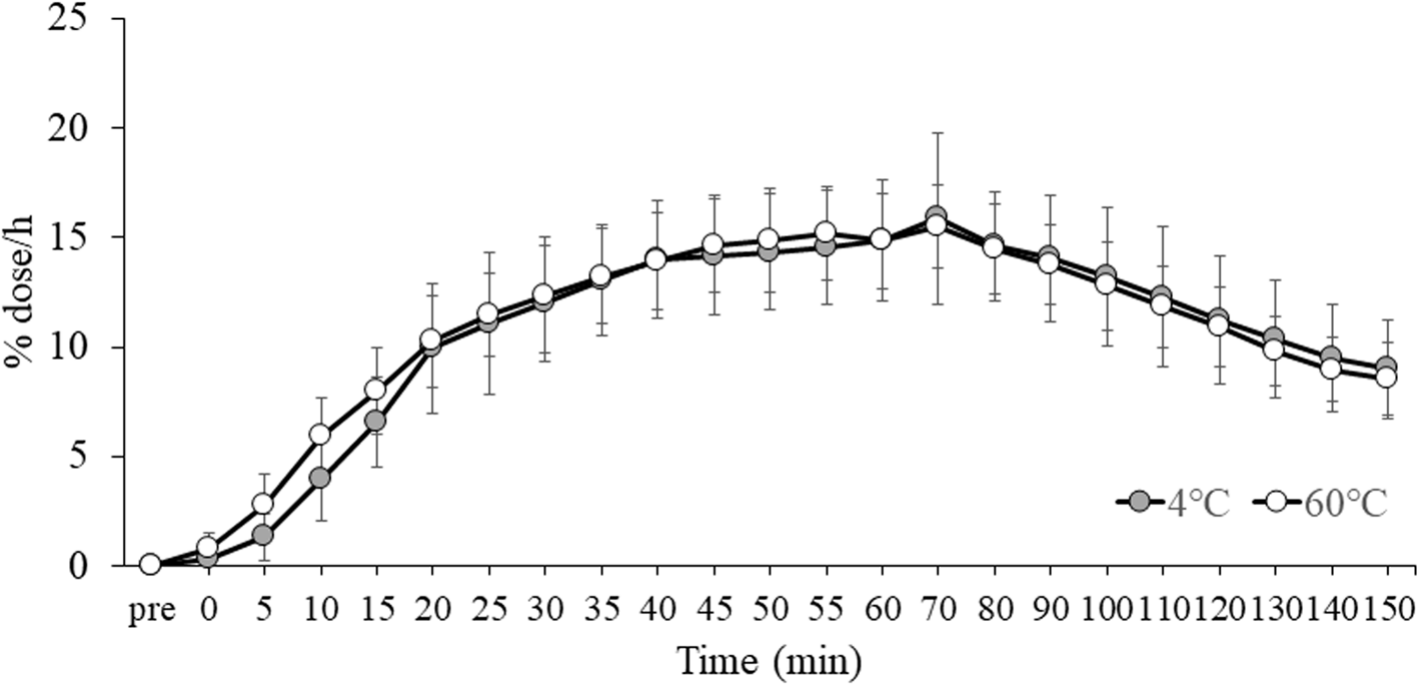
Excretion rate (%dose/h) values of.^13^CO_2_ over 150 min as shown in the [^13^C]-labeled acetic acid breath test at EX + 4 °C and EX + 60 °C trials. Data were analyzed using two-factor ANOVA. EX: 20 sets of moderate-intensity interval exercises, in which 1 set consisted of 120% maximum heart rate for 20 s and 25 W for 40 s; 4 °C: carbohydrate-protein-containing drink intake at 4 °C; 60 °C: carbohydrate-protein-containing drink intake at 60 °C. There was a significant main effect of time (*p* < 0.001). There were no main effect of trial (*p* = 0.463) and interaction (*p* = 0.352)

The values of %dose/h for the EX + 4 °C and EX + 60 °C trials from the start of the trial to 150 min after drink ingestion are shown in Fig. 2. There were no significant differences in %dose/h between the EX + 4 °C and EX + 60 °C trials from the start of the study until 150 min after drink ingestion (main effect of trial: ES = 0.061, *p* = 0.463; time: ES = 0.901, *p* < 0.001; interaction: ES = 0.112, *p* = 0.352) (There was also no significant difference in the AUC of %dose/h between EX + 4 °C and EX + 60 °C trials from the start of the study to 150 min after drink ingestion (95% CI: − 53.8 to 32.8, *p* = 0.606).

**Fig. 3:**
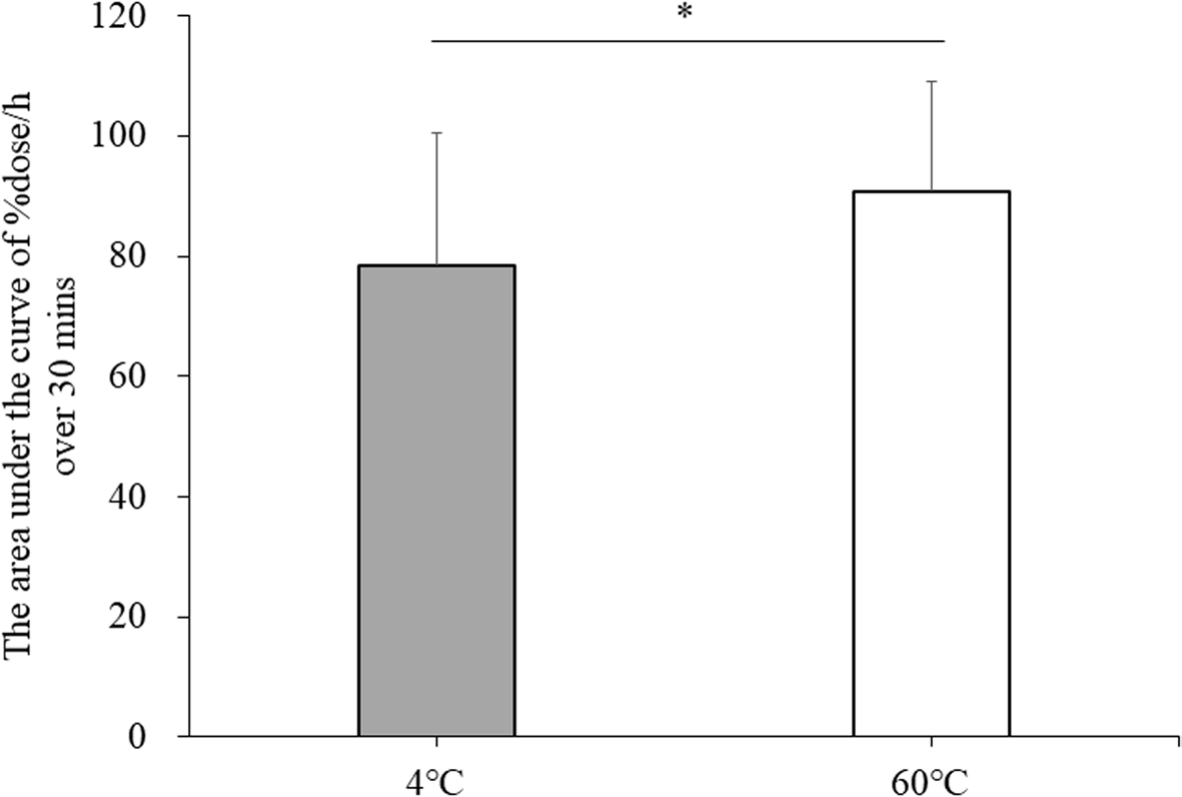
The AUC of %dose/h for the EX + 4 °C and EX + 60 °C trials from the start of the trial to 30 min after drink ingestion. Data were analyzed using *t*-test. EX: 20 sets of moderate-intensity interval exercises, in which 1 set consisted of 120% maximum heart rate for 20 s and 25 W for 40 s; 4 °C: carbohydrate-protein-containing drink intake at 4 °C; 60 °C: carbohydrate-protein-containing drink intake at 60 °C. There was a significant effect (*p* = 0.046). *p < 0.05

We focused on the data for 30 min after ingestion and analyzed them because differences in gastric emptying due to drink ingestion were observed up to 30 min after drink ingestion in a previous study [7]. Two-way ANOVA showed that the %dose/h values tended to be higher at EX + 60 °C than at EX + 4 °C from the start of the test until 30 min after drink ingestion (main effect of trial: ES = 0.277, *p* = 0.065; time: ES = 0.941, *p* < 0.001; interaction: ES = 0.183, *p* = 0.114). *T*-tests showed that the area under the curve (AUC) of %dose/h from the start of the trial to 30 min after the consumption of the drinks was higher at EX + 60 °C than at EX + 4 °C (95% CI: − 24.4 to − 0.3, *p* = 0.046). The AUC of %dose/h for the EX + 4 °C and EX + 60 °C trials from the start of the trial to 30 min after drink ingestion is shown in Fig. 3.

The mean values of T_max-calc_, T_1/2_, and %dose/h observed in each trial were compared to determine the effect of post-exercise carbohydrate-protein drink temperature on gastric emptying. The values of T_max-calc_ at 4 °C and 60 °C were 63.4 ± 14.7 min and 59.3 ± 13.2 min, respectively, and the values of T_1/2_ were 105.1 ± 19.9 min and 108.3 ± 24.8 min, respectively. There were no significant differences in the Tmax-calc (95% CI: − 3.1 to 11.4, *p* = 0.233) and T_1/2_ values between the EX + 4 °C and EX + 60 °C trials (95% CI: − 16.8 to 10.5, *p* = 0.618).

### Interstitial fluid glucose

**Fig. 4:**
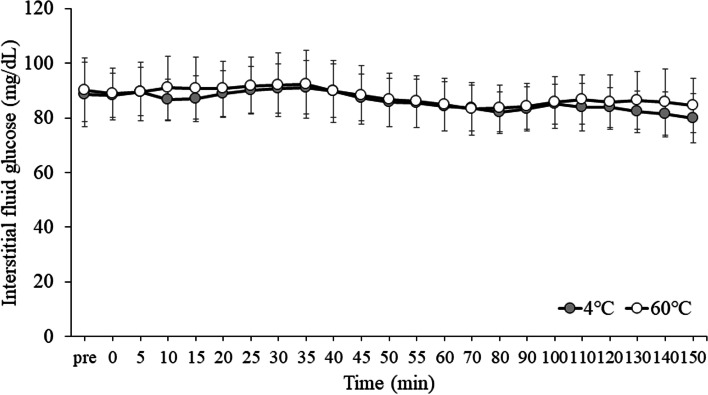
The values of interstitial fluid glucose over 150 min at EX + 4 °C and EX + 60 °C trials. Data were analyzed using two-factor ANOVA followed by a multiple comparison test using the Bonferroni method. EX: 20 sets of moderate-intensity interval exercises, in which 1 set consisted of 120% maximum heart rate for 20 s and 25 W for 40 s; 4 °C: carbohydrate-protein-containing drink intake at 4 °C; 60 °C: carbohydrate-protein-containing drink intake at 60 °C. There was a significant main effect of trial time (*p* < 0.001). There were no main effect of trial (*p* = 0.698) and interaction (*p* = 0.810)

The interstitial fluid glucose level from the start of the study to 150 min after drink ingestion is shown in Fig. 4. For the interstitial fluid glucose, there was a main effect of time (ES = 0.518, *p* < 0.001). There were no main effect of trial (*p* = 0.698) and interaction (*p* = 0.810). Post-hoc analysis indicated that glucose was lowered at 55 and 60 min compared to 30 min after consumption of the carbohydrate-protein-containing drink (55 min: *p* = 0.017, 60 min: *p* = 0.006). The total AUC values for interstitial fluid glucose from the start of the study to 150 min after drink ingestion did not differ between trials (3784.5 ± 351.7 mg/dL vs. 3861.9 ± 417.3 mg/dL for the EX + 4 °C and EX + 60 °C trials, respectively, ES = 0.005, *p* = 0.841). In addition, there was no significant difference in the AUC of %dose/h between EX + 4 °C and EX + 60 °C trials from the start of the study to 150 min after drink ingestion (95% CI: -120.9–100.1, *p* = 0.833).

### Appetite score and feelings of physical condition

The changes in appetite score and feeling of physical condition are shown in Table 3. Subjective gastrointestinal discomfort (i.e., “Does your stomach feel uncomfortable?”) showed a main effect of the trial and was lower at EX + 60 °C compared at EX + 4 °C (ES = 0.328, *p* = 0.041). There was no interaction between the trial and time (*p* = 0.564). There were no significant differences in subjective appetite perception (i.e., hunger, fullness, desire to eat, sweet, sour, fatty, and salty foods) and feelings of physical condition (i.e., “Do you feel exhausted?”) between trials.

**Table 3:** The changes in appetite score and feelings of physical condition

	EX + 4 °C								EX + 60 °C						p
	Pre	0	30	60	90	120	150	Pre	0	30	60	90	120	150	
**Perception of the appetite**															
Hunger (mm)	70.8 ± 10.9	46.4 ± 19.2	55.7 ± 12.1	61.9 ± 9.3	65.6 ± 15.6	74.3 ± 6.0	77.6 ± 5.0	69.9 ± 11.3	48.9 ± 20.1	49.3 ± 21.4	64.9 ± 18.0	70.0 ± 17.8	74.8 ± 14.8	78.3 ± 14.5	0.698
Fullness (mm)	22.5 ± 12.8	48.8 ± 23.7	40.9 ± 18.4	38.1 ± 15.6	31.1 ± 14.2	23.3 ± 8.5	19.9 ± 12.5	24.8 ± 20.0	56.5 ± 22.9	48.6 ± 19.4	33.0 ± 12.1	23.7 ± 12.1	25.1 ± 15.2	20.1 ± 12.0	0.699
Desire to eat (mm)	71.8 ± 8.2	57.3 ± 14.2	63.5 ± 14.4	67.5 ± 13.1	72.4 ± 8.0	76.9 ± 8.2	83.7 ± 6.5	67.3 ± 20.6	53.1 ± 23.2	57.2 ± 24.6	72.6 ± 20.0	77.3 ± 14.8	76.1 ± 15.7	81.3 ± 13.0	0.751
Desire to sweet foods (mm)	62.0 ± 17.2	53.3 ± 16.3	56.4 ± 12.7	62.7 ± 17.2	62.8 ± 14.5	69.8 ± 11.5	73.0 ± 10.7	58.9 ± 16.1	48.2 ± 13.7	55.1 ± 16.3	58.9 ± 16.5	60.5 ± 18.0	63.1 ± 20.2	65.7 ± 15.5	0.166
Desire to sour foods (mm)	38.9 ± 16.2	40.4 ± 18.1	41.9 ± 18.5	47.4 ± 20.1	44.8 ± 18.1	50.1 ± 22.5	48.8 ± 21.1	45.3 ± 16.2	44.8 ± 19.6	46.1 ± 20.0	45.5 ± 21.6	46.8 ± 21.3	46.3 ± 22.6	47.6 ± 21.6	0.738
Desire to fatty foods (mm)	44.2 ± 24.0	42.7 ± 17.7	52.4 ± 18.9	53.6 ± 24.9	57.3 ± 22.5	59.4 ± 24.4	60.7 ± 25.3	39.7 ± 21.9	43.8 ± 20.2	45.8 ± 24.1	50.5 ± 29.9	53.6 ± 24.4	55.3 ± 28.4	55.0 ± 28.4	0.352
Desire to salty foods (mm)	59.2 ± 13.4	65.3 ± 13.5	65.3 ± 11.5	67.8 ± 13.3	70.5 ± 9.0	71.7 ± 11.8	74.0 ± 10.8	55.8 ± 17.7	59.8 ± 13.1	62.3 ± 16.0	67.3 ± 17.1	66.2 ± 19.5	68.6 ± 17.0	71.4 ± 16.2	0.322
**Feelings of physical condition**															
Gastrointestinal discomfort (mm)	23.5 ± 21.8	21.6 ± 17.3	23.7 ± 19.8	20.8 ± 19.1	23.2 ± 22.6	26.6 ± 25.4	24.2 ± 23.5	22.8 ± 26.7	19.3 ± 16.7	22.9 ± 19.5	15.7 ± 15.3	17.0 ± 18.4	18.8 ± 19.2	15.8 ± 16.5	0.041
Physical exhaustion (mm)	32.7 ± 21.9	55.3 ± 10.6	51.9 ± 18.8	44.3 ± 17.4	39.8 ± 20.0	43.8 ± 21.4	39.0 ± 26.6	36.7 ± 22.1	61.6 ± 14.6	46.4 ± 11.6	40.5 ± 19.0	37.3 ± 19.5	31.8 ± 15.2	29.9 ± 14.7	0.427

*N* = 12; data are means ± SD. Data were analyzed using two-way ANOVA. EX: 20 sets of moderate-intensity interval exercises, in which 1 set consisted of 120% maximum heart rate for 20 s and 25 W for 40 s; 4 °C: carbohydrate-protein-containing drink intake at 4 °C; 60 °C: carbohydrate-protein-containing drink intake at 60 °C

## Discussion

This study demonstrated that consuming of a carbohydrate-protein-containing drink at 60 °C after intermittent cycling exercise slightly accelerated gastric emptying over the following 30 min, compared to consuming of a carbohydrate-protein-containing drink at 4 °C. In addition, the present study showed that consuming of a carbohydrate-protein-containing drink at 60 °C reduced subjective gastric discomfort following consumption of the drink compared to consuming of a carbohydrate-protein-containing drink at 4 °C. The present study is the first to examine how different temperatures of carbohydrate-protein-containing drinks after exercise affect the gastric emptying rate and interstitial fluid glucose in healthy humans. Our findings demonstrate the drink temperature may be one of the factors that influence gastric emptying and gastric discomfort after exercise.

The effect of the temperatures of nutritional drink consumption at rest on the rate of subsequent gastric emptying has been disparate among four laboratory-based studies [6–9]. Mishima et al. examined the gastric emptying of liquid and solid meals (200 mL) at 4 °C, 37 °C, and 60 °C in 25 healthy volunteers using the ^13^C breath test, and found that liquid and solid meals at 60 °C accelerated gastric emptying compared with 37 °C during the initial 30 min [7]. The result that the percent excretion of ^13^CO_2_ tended to be higher in the EX + 60 °C trial compared to EX + 4 °C trial in the present study confirms the results of the resting study by Mishima et al. [7]. While gastric temperature may be higher after exercise than at rest as the core temperature is temporarily elevated, the effect of drink temperature on gastric emptying rate may not differ between rest and after 20 min of intermittent exercise. Other studies [6, 8, 9] have shown that drinks at temperatures higher or lower than body temperature delayed gastric emptying. Differences in the methods of measuring gastric emptying and the nutrient composition of the drinks may explain the discrepancy between our results and those of the present study.

No study has examined the effect of post-exercise nutrient temperature on gastric emptying, and the results may have differed according to the different digestion and absorption conditions at rest or after exercise. Several previous studies have demonstrated that the rate of gastric emptying after exercise is influenced by the nutrient composition of the drinks [27, 28] and timing of ingestion [2–4], as well as by the intensity of the exercise [2, 4, 29, 30]. Previous studies have shown that post-exercise gastric emptying is delayed with carbohydrate drinks rather than water [27] and with whey protein rather than maltodextrin drinks [28]. Gastric emptying was also delayed immediately after exercise compared with 30 min after exercise [2, 3], and there is no consensus on whether gastric emptying varies with exercise intensity and type [2, 4, 29, 30]. Gastric emptying rate was delayed 5 min and 30 min after exercise in intermittent exercise with cycling at 120% maximal oxygen uptake followed by 20 W [2]. In contrast, after exercise, no difference in gastric emptying rate was reported between 5 and 30 min after a single bout of 30 min of mild–moderate-intensity cycling exercise with a heart rate of approximately 120 bpm [4]. Although the present study could not address whether gastric emptying was altered by moderate-intensity intermittent exercise, it showed that gastric emptying after exercise might be varied with drink temperature.

Carbohydrates and proteins ingested after exercise are then ejected from the stomach, following which they are then broken down by enzymes into monosaccharides and amino acids/dipeptides/tripeptides, and absorbed in the small intestine to facilitate muscle recovery. Drink temperature may not influence the rate of absorption, as interstitial fluid glucose levels did not change with different temperatures of drink. Although the present study used interstitial fluid glucose levels to measure the glucose concentration continuously, a previous study reported a gap between blood glucose levels and interstitial fluid glucose levels during the post-exercise period [23]. Since glucose initially enters the blood and subsequently enters the interstitial fluid, there is a time lag between blood glucose levels and interstitial fluid glucose levels [23]. Although interstitial fluid glucose levels did not vary with drink temperatures in the present study, this might be due to the properties of the interstitial fluid glucose and should be considered preliminary.

Changes in gastric motility via intragastric temperature changes may explain the slight differences in gastric emptying rate. Sun et al. found that the intragastric temperature warmed to about 43 °C after ingesting orange juice at 50 °C [6]. It is expected that the intragastric temperature was temporarily raised in the EX + 60 °C trial in the present study. Temperature changes in the stomach are sensed by the activation of thermoreceptors [31]. The activity of thermoreceptors is associated with gastric motility [32]. TRPV2, activated by thermal stimulation over 52 °C, promotes gastric emptying by activation [33]. In contrast, TRPM8 and TRPA1 may have been activated during the EX + 4 °C trial. In a study examining changes in TRPM8 receptor activation and gastric contractions by gastric cooling in rats, cooling to 15 °C increased gastric contractions, whereas stimulation at lower temperatures decreased gastric contractions [34]. TRPA1, activated at 17 °C, has been shown to delay gastric emptying in vivo via serotonergic pathways [35]. According to these studies, TRPV2, TRPM8, and TRPA1 may explain the differences in gastric emptying by drink temperatures.

Changes in gastric motility via changes in intragastric temperature may explain the slight differences in gastric emptying rate by drink temperature. Sun et al. observed the changes in intragastric temperature when 400 mL of orange juice was consumed at 4 °C and 50 °C and found that the intragastric temperature took longer to return to the normal temperature (37 °C) for the 4 °C drink compared to at 50 °C [6]. Changes in gastric temperature are perceived by the activation of thermoreceptors [31]. The activity of gastric thermoreceptors has been shown to be related to gastric motility [32], which may have been altered by a change in intragastric temperature after consuming the drink in the EX + 60 °C trial.

Indeed, the consumption of hot drinks after exercise has been reported to increase gastric motility. The authors reported that consumption of a protein-containing drink at 60 °C after running exercise at 80% of maximum heart rate increased gastric pyloric contractions compared to a 2 °C trial [13]. Gastric motility and gastric emptying rate are closely related [36], and in the EX + 60 °C trial, gastric motility was enhanced after consumption of the hot drink, which may have accelerated the rate of gastric emptying. In the present study, gastric discomfort after drink consumption was lower in the EX + 60 °C trial compared to that in the EX + 4 °C trial. As gastric motility is temporarily reduced after the consumption of cold drinks [12, 13], the gastric fullness and discomfort may have been maintained in the EX + 4 °C trial. According to the results of these studies, the temperature of the drinks consumed after exercise could affect gastric discomfort via changes in gastric motility. However, these results may be limited to carbohydrate-protein-containing drinks that contain lactose. Although the carbohydrate-protein-containing drinks used in the present study contain lactose, the optimum temperature for lactase, which is necessary for the decomposition of lactose, is approximately body temperature and may be inhibited at low temperatures. It is possible that subjects felt gastric discomfort after consuming the drinks at 4 °C in the present study due to the inhibition of lactase activity.

The present study has several strengths. This study is the first to quantitatively evaluate the effects of the drink temperature on gastric emptying after exercise. Previously, post-exercise nutrition has been discussed in terms of drink composition, quantity [37] and timing of consumption [38]. The present study revealed the effect of the temperature of post-exercise drinks on digestion, which may contribute to the establishment of strategies for rapid nutrient intake after exercise. Moreover, the present study investigated appetite and gastric discomfort using the VAS to assess the effects of post-exercise nutritional drink temperature on subjective gastrointestinal symptoms, along with objective measures such as gastric emptying rate and interstitial fluid glucose levels. The present results, i.e., a reduction in gastric discomfort when consuming hot drinks after exercise, will contribute to the selection of nutritional drinks for sports situations.

The present study has several limitations. First, this study did not measure changes in core temperature due to post-exercise drink ingestion. Since exercise increases core temperature [15], the effects of drink temperature on digestive function might be changed. Future studies should track the core temperature to assess the effect of drink temperature on digestive function. In addition, this study used interstitial fluid glucose levels to assess blood glucose uptake after ingestion of a carbohydrate-protein-containing drink. To assess physical recovery after exercise, evaluation of direct muscle glycogen and muscle protein recovery is recommended.

## Conclusions

In conclusion, consuming of carbohydrate-protein-containing drink at 60 °C after intermittent cycling tended to accelerate gastric emptying during the first 30 min after consuming the drink and to reduce gastric discomfort during the last 150 min after consuming the drink, compared with consuming of carbohydrate-protein drink at 4 °C after intermittent cycling in healthy young men. Drink temperature might be one of the factors that determine the rate of gastric emptying and gastric discomfort after intermittent exercise.

## Data Availability

Not applicable.

## Abbreviations

RPE: Ratings of perceived exertion
HR: Heart rate
VAS: Visual analog scale
Tmax-calc: The time of maximum [^13^CO_2_] emissions per hour
T_1/2_: The time of half [^13^CO_2_] emissions per hour
%dose/h: The value of ^13^CO_2_ excretion rate in 1 h
ANOVA: Analysis of variance
ES: Effect sizes
95% CI: The 95% confidence intervals
